# Syntheses, crystal structure and thermal behavior of poly[di-μ-bromido-μ-2,3-di­methyl­pyrazine-κ^2^*N*^1^:*N*^4^-cadmium(II)]

**DOI:** 10.1107/S205698902600736X

**Published:** 2026-07-23

**Authors:** Christian Näther

**Affiliations:** a Institut für Anorganische Chemie, Universität Kiel, Max-Eyth.-Str. 2, 24118 Kiel, Germany; University of Aberdeen, United Kingdom

**Keywords:** coordination polymer, layered compound, synthesis, cadmium bromide, 2,3-di­methyl­pyrazine, crystal structure

## Abstract

The title compound, [CdBr_2_(C_6_H_8_N_2_)]_*n*_, was prepared by the reaction of cadmium bromide with 2,3-di­methyl­pyrazine in aceto­nitrile. Powder X-ray diffraction (PXRD) indicated that a pure phase had been obtained. The asymmetric unit consists of one Cd cation located on a center of inversion, one 2,3-di­methyl­pyrazine ligand situated on a twofold rotation axis and one bromide anion in a general position. In the extended structure, the cadmium cation is octa­hedrally coordinated by four bridging bromide anions and two bridging 2,3-di­methyl­pyrazine coligands. The cations are linked *via* common bromide-ion edges into [010] chains that are further connected into (100) layers by the bridging 2,3-di­methyl­pyrazine coligands. Measurements using thermogravimetry and differential thermoanalysis reveal that the compound decomposes in two separate steps in which a more 2,3-di­methyl­pyrazine-deficient compound is formed, which according to PXRD measurements is crystalline.

## Abstract 2

In the crystal structure of the title compound, the cadmium cations are linked by pairs of μ-1,1 bridging bromide anions into chains that are further connected into layers by the bridging 2,3-di­methyl­pyrazine coligands.

## 1. Chemical context

For many years, we and others have been inter­ested in the crystal structures of transition-metal halide and pseudohalide coordination compounds because they show a large structural variability, which frequently leads to the formation of different metal halide substructures (Kromp & Sheldrick, 1999 ▸; Peng *et al.*, 2010 ▸; Li *et al.*, 2005 ▸; Näther *et al.*, 2002 ▸). In the beginning, we focused on compounds with copper(I) halides and pseudohalides and later this was expanded to divalent cations like zinc or cadmium. What is common to all of these compounds is the fact that for a given metal halide and a given coligand, in most cases compounds of different stoichiometry can be obtained. If this is the case, the coligand-deficient compounds can frequently be detected and prepared by thermal ligand removal, because the coligands are usually lost in separate steps (Näther *et al.*, 2001 ▸; Näther & Jess, 2001 ▸).

In the course of our project we became inter­ested in zinc and cadmium halide coordination compounds with 2,3-di­methyl­pyrazine (C_6_H_8_N_2_) as coligand. With CdI_2_ a coligand-rich compound with the composition CdI_2_(C_6_H_8_N_2_)_2_ and a coligand-deficient compound with the composition CdI_2_(C_6_H_8_N_2_) were obtained (Näther, 2026*a* ▸). The first compound consists of discrete complexes with cadmium in a tetra­hedral environment. In the second compound, the cadmium cations are also tetra­hedrally coordinated and linked by the 2,3-di­methyl­pyrazine ligands into chains. These structures are therefore very similar to those of the corresponding compounds with zinc halides (Näther & Bhosekar, 2025*a* ▸,*b* ▸, 2026 ▸; Yang *et al.*, 2025 ▸). These structures with CdI_2_ are somehow surprising because, in contrast to zinc, an octa­hedral coordination is usually preferred for cadmium halide coordination compounds. Therefore, for these compounds structures were expected in which the Cd cations are linked by pairs of iodide anions into chains. In this case, the 2,3-di­methyl­pyrazine coligand would be terminally coordinated in CdI_2_(C_6_H_8_N_2_)_2_, whereas in CdI_2_(C_6_H_8_N_2_) the coligand would act as a bridging ligand. This is exactly the case for the corresponding compounds with, for example, pyrazine, 2-methyl­pyrazine and 2-chloro­pyrazine as coligand (Bailey & Pennington, 1997 ▸; Pickardt & Staub, 1997 ▸; Näther *et al.*, 2017 ▸).

Based on these findings, we tried to prepare the corresponding compounds with cadmium bromide and we accidentally obtained crystals of a solvate with the composition CdBr_2_(C_6_H_8_N_2_)(H_2_O)·C_6_H_8_N_2_·0.5H_2_O, in which the ratio between CdBr_2_ and coordinating coligands is 1:2 (Näther, 2026*b* ▸). In contrast to the corresponding compounds with CdI_2_, in this structure CdBr_2_ chains occur and an octa­hedral coordination of the cadmium cations is observed. Therefore, one can assume that a more coligand-deficient compound with the composition CdBr_2_(C_6_H_8_N_2_) might exist, in which similar CdBr_2_ chains are connected by the 2,3-di­methyl­pyrazine ligands into layers. To investigate this assumption, additional synthetic investigations were performed in which crystals of the title compound were obtained, which were characterized by single crystal X-ray diffraction.
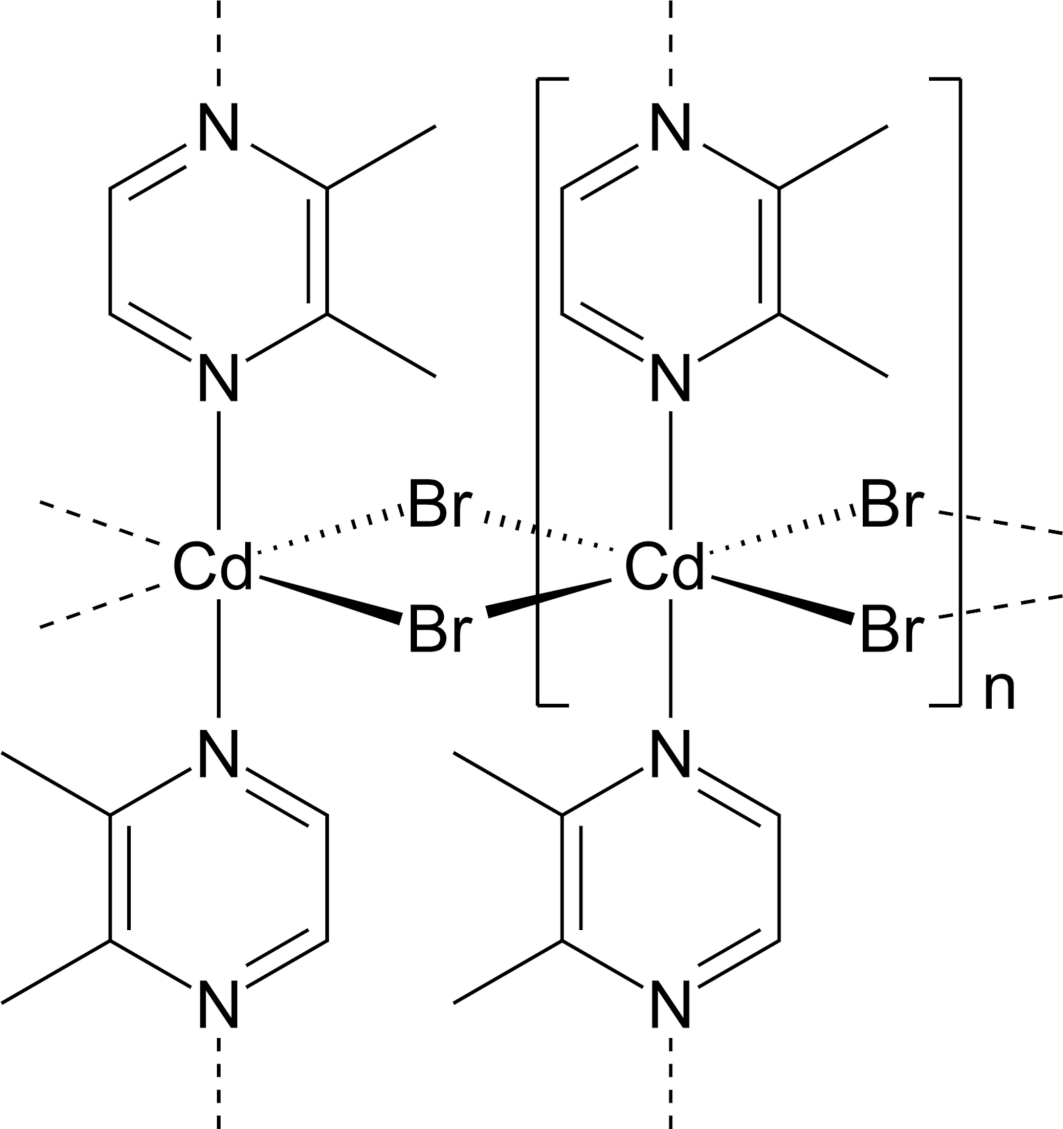


## 2. Structural commentary

**Figure 1:**
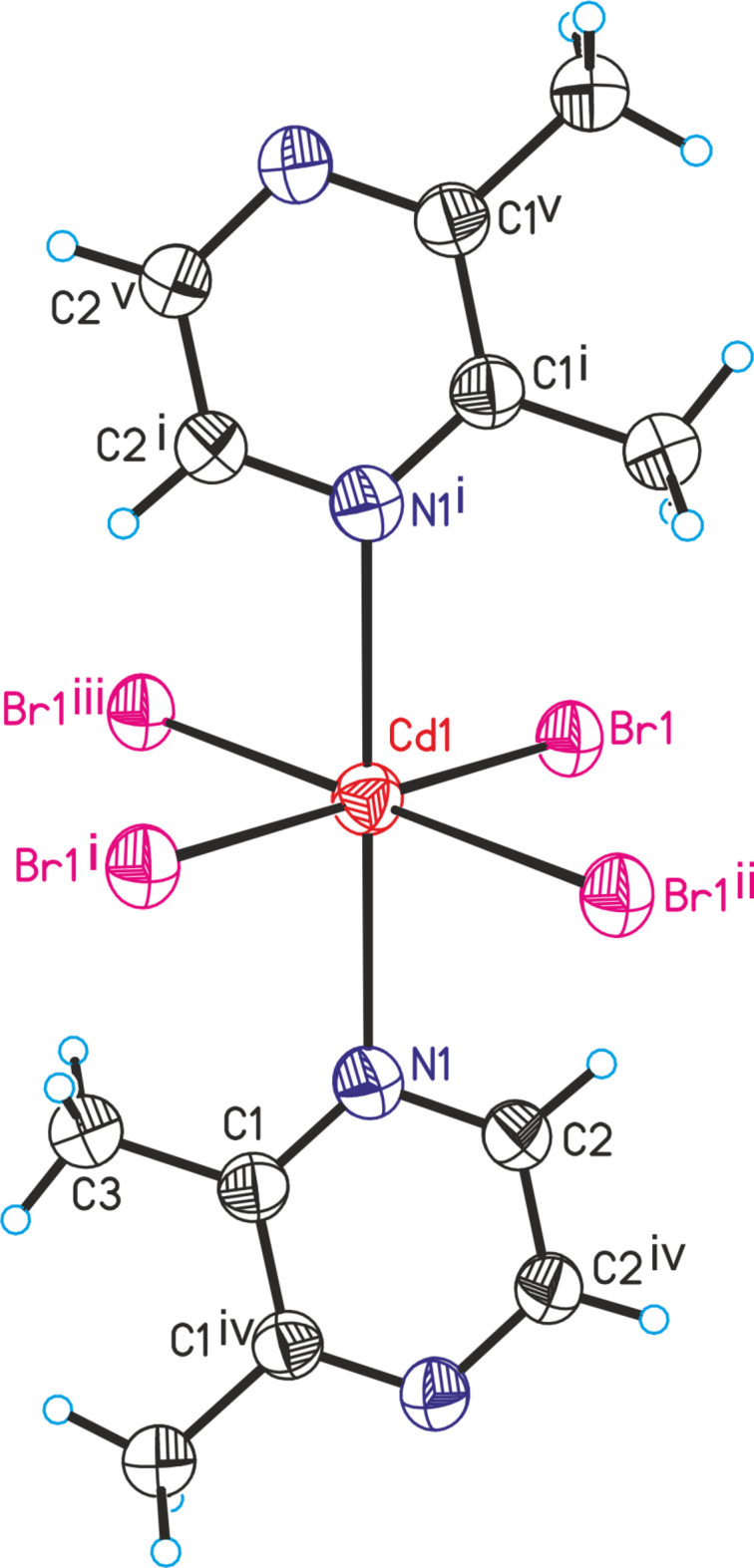
The asymmetric unit of (**I**) expanded to show the complete ligands and cadmium coordination polyhedron with displacement ellipsoids drawn at the 50% probability level. Symmetry codes: (i) −*x* + 1, −*y* + 1, −*z* + 1; (ii) *x* + 1, *y*, *z*; (iii) −*x*, −*y* + 1, −*z* + 1; (iv) −*x* + 1, *y*, −*z* + 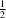; (v) *x*, *y* + 1, *z* + 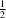.

**Figure 2:**
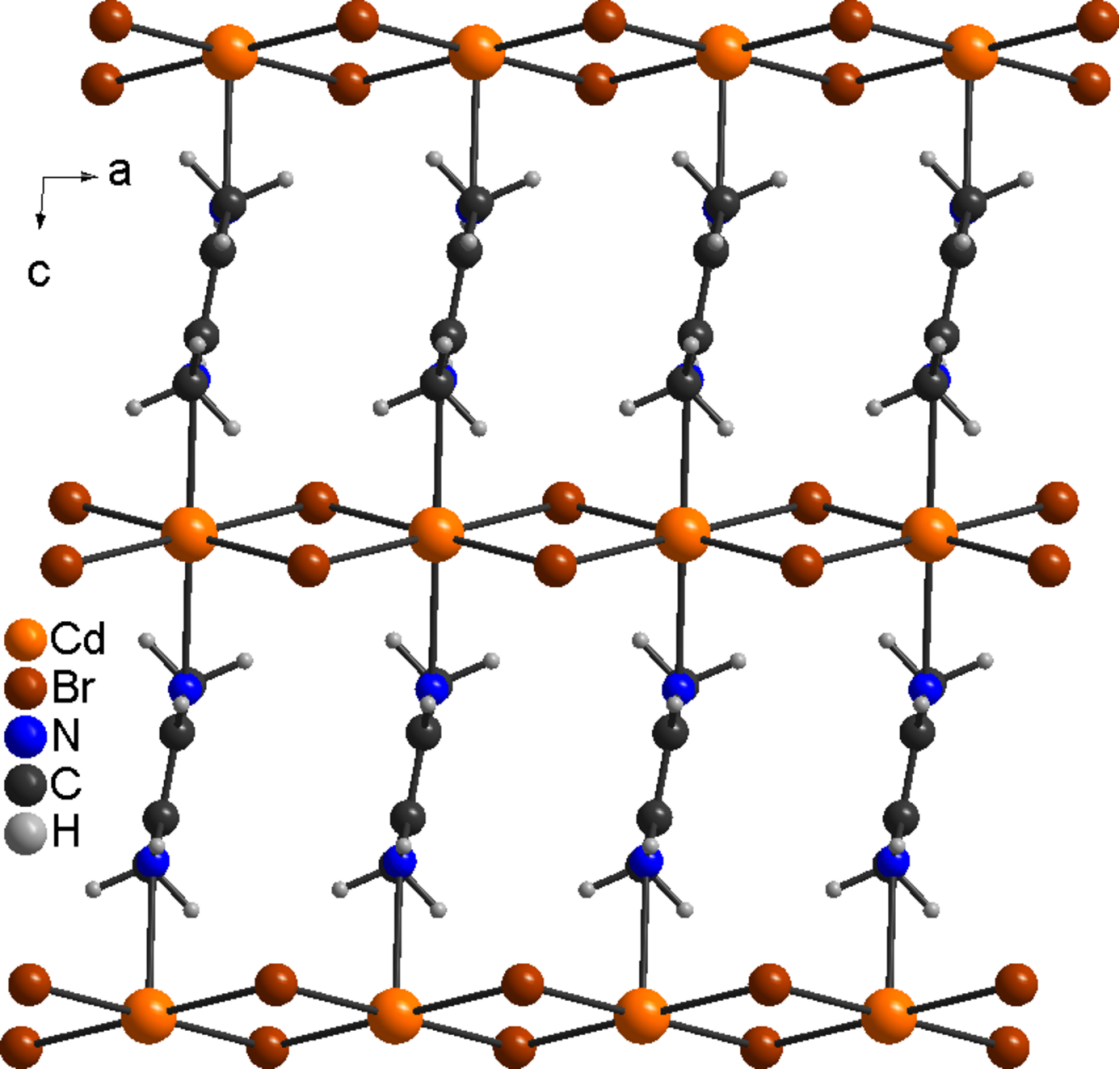
View of a part of a chain in the crystal structure of (**I**).

The asymmetric unit of the title compound, CdBr_2_(C_6_H_8_N_2_) (C_6_H_8_N_2_ = 2,3-di­methyl­pyrazine) (**I**) (Fig. 1 ▸), is built up from one cadmium cation that occupies a center of inversion, one 2,3-di­methyl­pyrazine ligand generated by a twofold rotation axis bis­ecting the pairs of ring carbon atoms, and one bromide anion in a general position. Crystal symmetry leads to the cadmium cations being sixfold coordinated by four bromide anions that are located in the basal plane and two 2,3-di­methyl­pyrazine ligands in the apical positions. Bond angles deviate from the ideal values (Table 1 ▸), which shows that the Cd cation is in a slightly distorted octa­hedral environment (Table 1 ▸). The cations are linked by pairs of μ-1,3 bridging bromide anions into linear chains that propagate in the crystallographic *b*-axis direction (Fig. 2 ▸). These chains are built up from octa­hedra that share common bromide-ion edges [Br⋯Br = 3.760 (2) Å]. The bridging co-ligands connect the chains into (100) layers.

This structure is therefore completely different from that of CdI_2_(C_6_H_8_N_2_) (Näther, 2026*a* ▸) in which the cadmium cations are in a tetra­hedral environment, which might be more stable because of repulsion between the bulky iodide anions. However, there are a number of compounds with other pyrazine derivatives reported that show the same topology of the coordination network as that in the title compound. This also includes compounds with CdI_2_, which shows that the situation is more complex. With pyrazine as ligand this includes Cd*X*_2_(pyrazine) (*X* = Cl, Br, I; CSD refcodes TISSUJ, RINSIQ, RINSOW, Bailey & Pennington, 1997 ▸; RINSOW01, Pickardt & Staub, 1997 ▸; RINSOW02, Niu *et al.*, 2005 ▸). This also includes compounds with 2-methyl­pyrazine as ligand such as the isotypic compounds Cd*X*_2_(2-methyl­pyrazine) (*X* = Cl, Br, QAWHEE and QAWHUU, Näther *et al.*, 2017 ▸) and CdI_2_(2-methyl­pyrazine) (QAWHAA, Näther *et al.*, 2017 ▸).

## 3. Supra­molecular features

**Figure 3:**
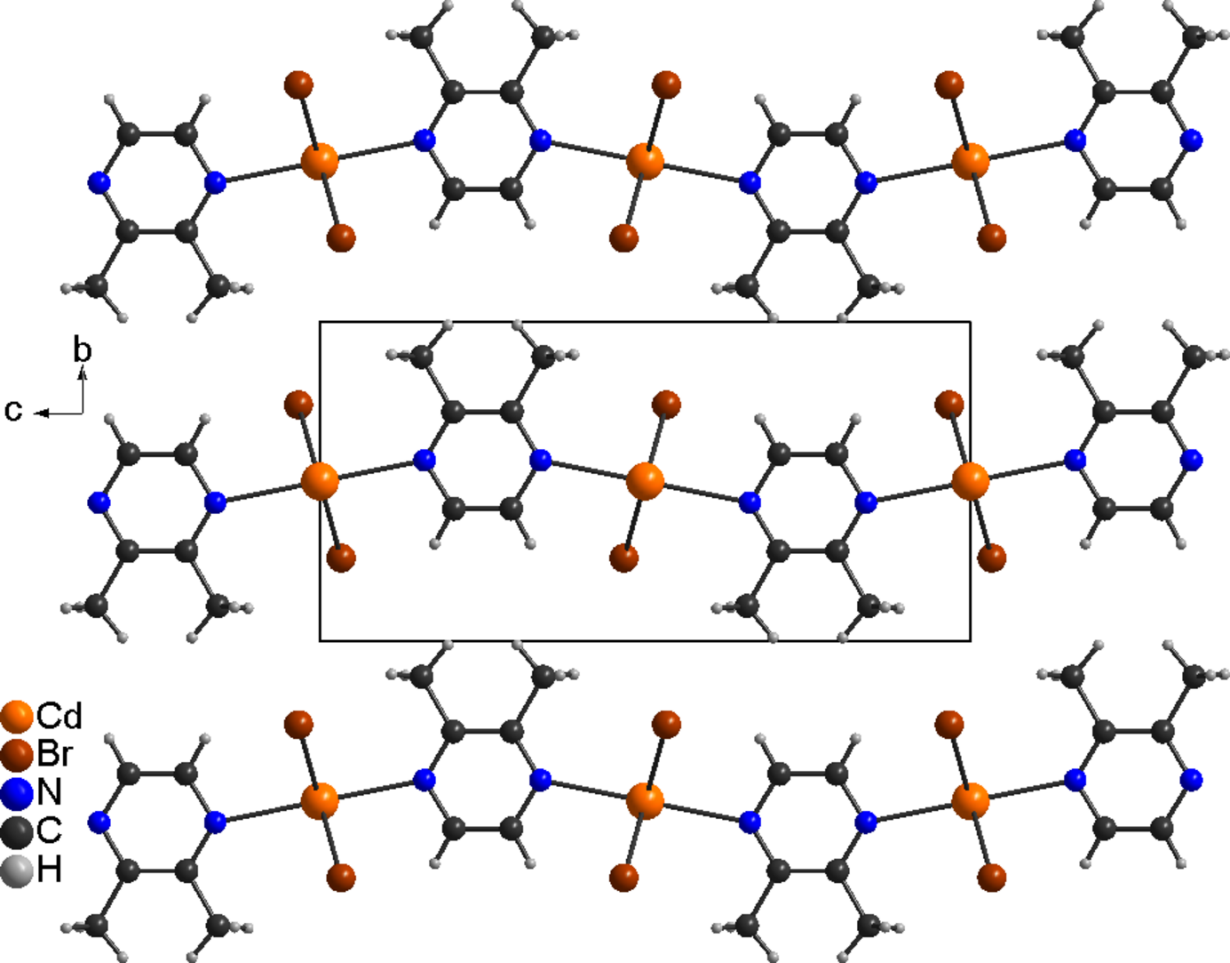
Crystal structure of (**I**) with view along the crystallographic *a*-axis direction.

In the extended structure of (**I**), the layers are stacked perpendicular to the crystallographic *a*-axis direction (Fig. 3 ▸). There are two C—H⋯Br contacts with one of them at a long distance (H⋯Br = 3.03 Å) and an angle far from linearity, indicating a very weak inter­action. The second one between a methyl hydrogen atom and the bromide atom is shorter with an angle close to linearity, indicating a stronger inter­action (Table 2 ▸).

## 4. Additional characterization

**Figure 4:**
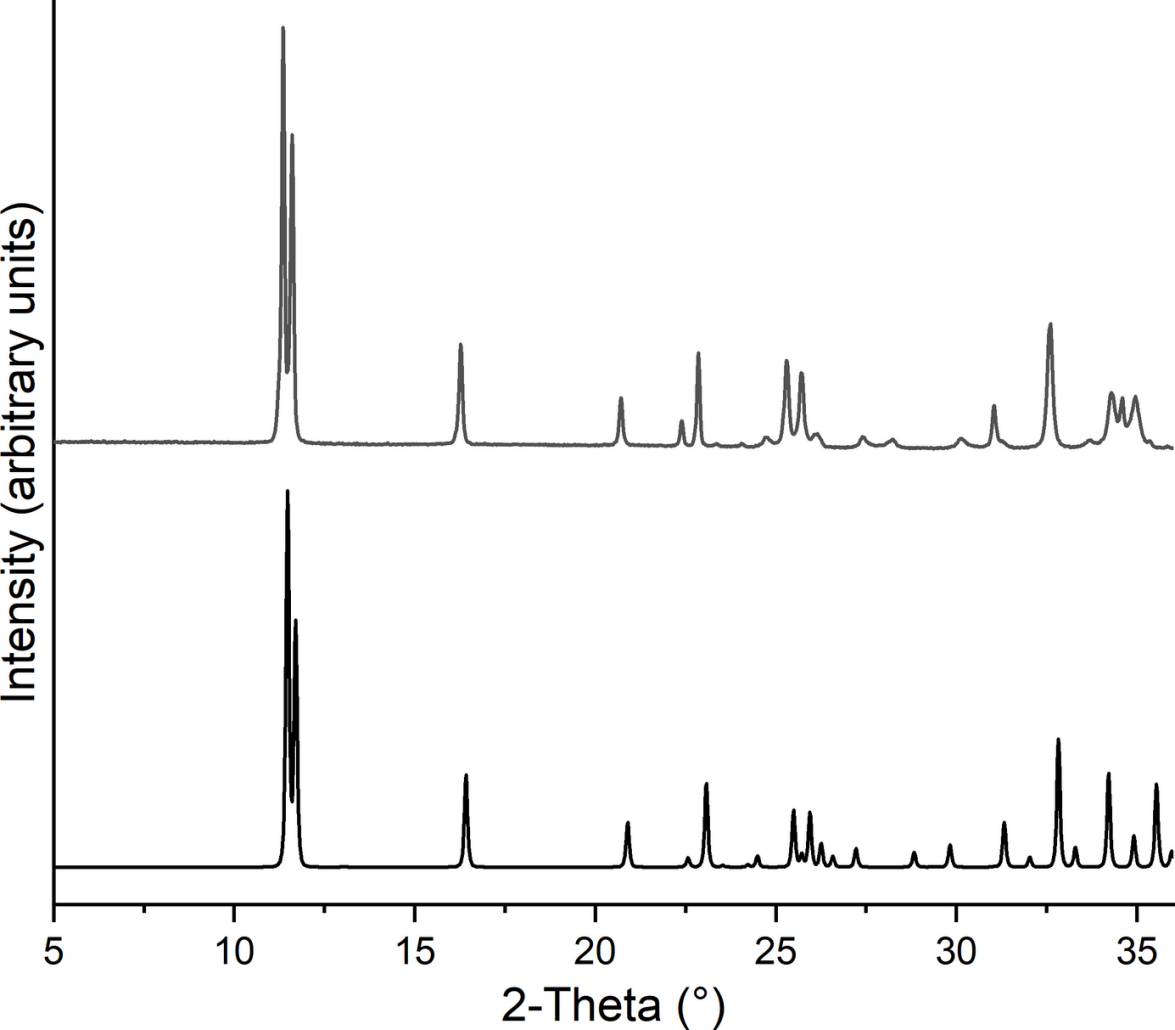
Experimental (top) and calculated (bottom) powder patterns for the title compound.

**Figure 5:**
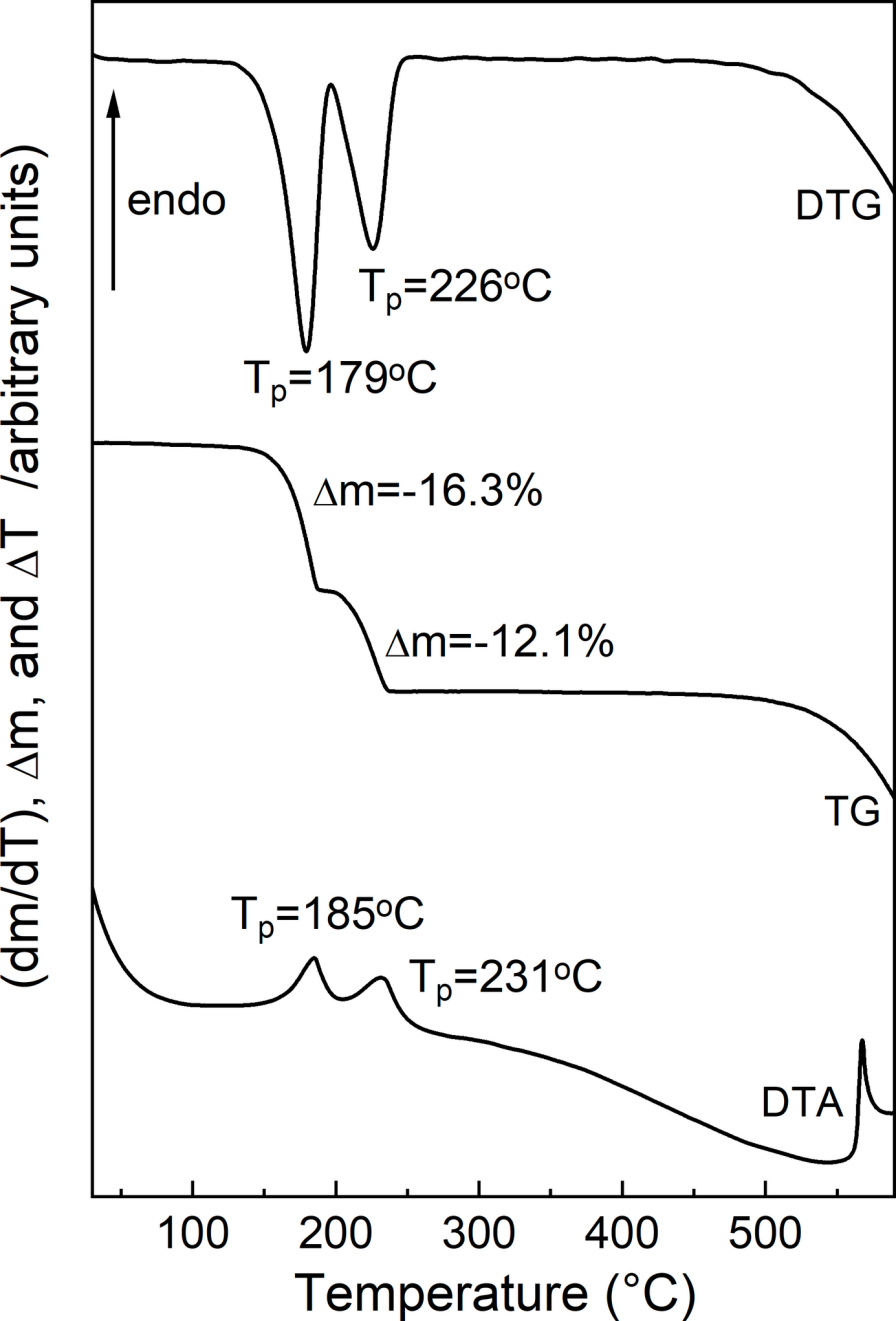
DTG, TG and DTA curves for the title compound.

**Figure 6:**
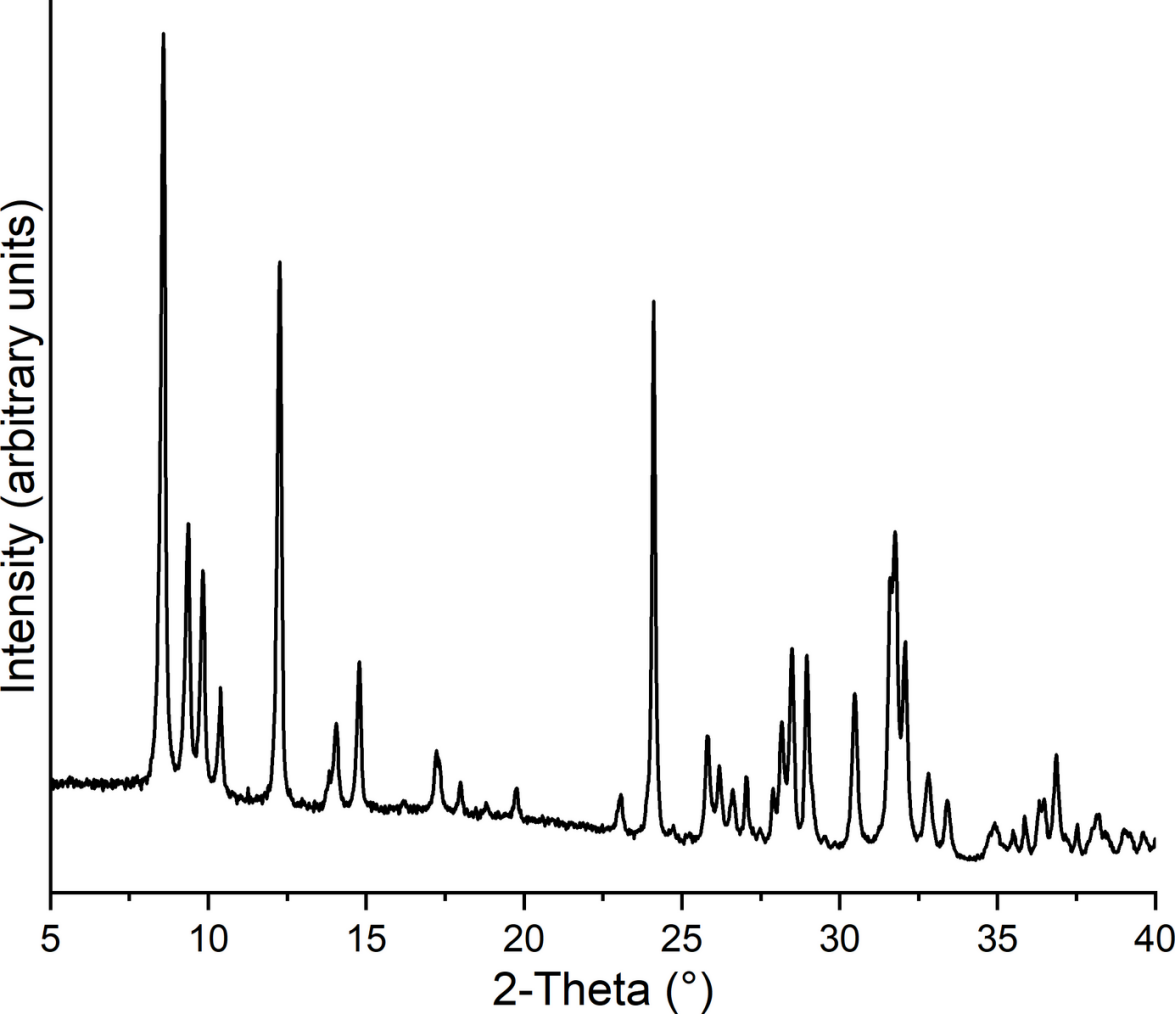
Experimental powder pattern of the residue obtained after the first mass loss in a TG measurement of the title compound.

Comparison of the experimental X-ray powder pattern of (**I**) with that calculated from single-crystal data shows that the title compound has been obtained as a pure phase (Fig. 4 ▸). Measurements using thermogravimetry coupled to differential thermoanalysis show that the title compound decomposes in two separate and endothermic steps, in which a 2,3-di­methyl­pyrazine deficient compound is formed as an inter­mediate (Fig. 5 ▸). PXRD data show that this inter­mediate is of reasonable crystallinity (Fig. 6 ▸). However, the experimental mass loss is not in good agreement with that calculated for the removal of half of the 2,3-di­methyl­pyrazine ligands and therefore, conclusions concerning the composition of this new crystalline phase cannot be made. In this context it is noted that compounds are reported that exhibit an unusual ratio between the cadmium halide and the organic coligands as in *catena*[hexa­kis­(μ_3_-chloro)­tetra­kis­(μ_2_-chloro)­bis­[μ_2_-1,2-bis­(1*H*-benzotriazol-1-yl)ethane]­penta­cadmium]] with a ratio of 2:5 (EMOWAF, Zhai *et al.*, 2011 ▸). In any case this inter­mediate compound must have a more condensed CdI_2_ network with μ_3_-bridging bromide anions.

## 5. Database survey

As already mentioned in the *Chemical context* section, some compounds with zinc halides and 2,3-di­methyl­pyrazine are known, and in all of them the zinc cations are tetra­hedrally coordinated. This coordination is also found in the cadmium iodide coordination compounds with 2,3-di­methyl­pyrazine as coligand whereas in the only known compound with CuBr, an octa­hedral coordination and CuBr chains are observed and this was the origin of the present work.

It was also mentioned that many compounds with other pyrazine derivatives and cadmium halide chains found in the Cambridge Structural Database (CSD Version 5.43, 2025; Groom *et al.*, 2016 ▸) using CONQUEST (Bruno *et al.*, 2002 ▸) are built up of Cd*X*_2_ chains, indicating that an octa­hedral coordination is preferred. That the situation is more complex is obvious from a search for Cd*X*_2_ compounds with similar but monocoordinating ligands, and pyridine and its derivatives would be representative examples. With pyridine, many compounds with cadmium halides are reported in which the ratio between Cd*X*_2_ and pyridine is 1:2. For such compounds, both a tetra­hedral and an octa­hedral coordination is found. CdCl_2_(pyridine)_2_ consists of CdCl_2_ chains with octa­hedral cadmium cations (CDPYCL01; Paulus, 1969 ▸, CDPYCL03; Satoh *et al.*, 2001 ▸ and CDPYCL04; Hu *et al.*, 2003 ▸). The same structure is found for CdBr_2_(pyridine)_2_, which crystallizes in two different polymorphic modifications (Hu *et al.*, 2003 ▸). In contrast, for CdI_2_(pyridine)_2_, discrete complexes with a tetra­hedral coordination of the Cd cations are observed (IPPAYAZ01; Hu *et al.*, 2003 ▸). Similar structures are found with 3-methyl­pyridine. CdCl_2_(3-methyl­pyridine)_2_ (BASQOC; Satoh *et al.*, 2001 ▸ and BASQOC01; Hu *et al.*, 2003 ▸) and CdBr_2_(3-methyl­pyridine)_2_ (IPAZOO, Hu *et al.*, 2003 ▸) show the same chain structure, while CdI_2_(3-methyl­pyridine)_2_ (IPEBAG; Hu *et al.*, 2003 ▸) consists of discrete tetra­hedral complexes. Finally, the corresponding compounds with 2-methyl­pyridine, CdBr_2_(2-methyl­pyridine)_2_ (EYEROQ; Bowmaker *et al.*, 2011 ▸) and CdI_2_(2-methyl­pyridine)_2_ (EYESOR; Bowmaker *et al.*, 2011 ▸), crystallize as discrete complexes, while the structure of the chloride compound is unknown. For the latter compounds, this might be traced back to steric repulsion between the metal cations and the methyl group in an octa­hedral coordination. This also suggests that for the iodide compound with pyridine derivatives, the formation of discrete complexes originates from steric reasons because of the much larger radii of iodide anions but it must be kept in mind that in CdI_2_(pyrazine) (RINSOW; Bailey & Pennington, 1997 ▸), a chain structure is found.

## 6. Synthesis, crystallization and experimental details

Cadmium bromide and 2,3-di­methyl­pyrazine were purchased from Sigma-Aldrich: 0.5 mmol (136.1 mg) CdBr_2_ and 0.5 mmol (54.1 mg) 2,3-di­methyl­pyrazine were stirred in 2 ml of aceto­nitrile for 2 d at room-temperature. Without stirring, single crystals of (**I**) in the form of colorless blocks were obtained within 3d.

The PXRD measurements were performed with Cu *K*α_1_ radiation (λ = 1.540598 Å) using a Stoe Transmission Powder Diffraction System (STADI P) equipped with a MYTHEN 1K detector and a Johansson-type Ge(111) monochromator.

Thermogravimetry and differential thermoanalysis (TG-DTA) measurements were performed in a dynamic air atmosphere in Al_2_O_3_ crucibles using a STA-PT 1000 thermobalance from Linseis. The instrument was calibrated using standard reference materials.

## 7. Refinement

Crystal data, data collection and structure refinement details are summarized in Table 3 ▸. The hydrogen atoms were positioned with idealized geometry (methyl H atoms allowed to rotate but not to tip) and were refined as riding atoms with *U*_iso_(H) = 1.2 *U*_eq_(C) (1.5 for methyl H atoms). The crystal chosen for data collection was found to be twinned. Both domains were indexed separately and finally a twin refinement using data in HKLF-5 format was performed leading to an BASF parameter of 0.212 (4).

## Supplementary Material

Crystal structure: contains datablock(s) I. DOI: 10.1107/S205698902600736X/hb8231sup1.cif

Structure factors: contains datablock(s) I. DOI: 10.1107/S205698902600736X/hb8231Isup2.hkl

CCDC reference: 2574160

Additional supporting information:  crystallographic information; 3D view; checkCIF report

## Figures and Tables

**Table 1:** Selected geometric parameters (Å, °)

Cd1—N1	2.531 (4)	Cd1—Br1^i^	2.7741 (5)
Cd1—Br1	2.6789 (5)		
			
N1—Cd1—Br1^ii^	91.66 (10)	N1^ii^—Cd1—Br1^i^	94.08 (10)
N1—Cd1—Br1	88.34 (10)	Br1^ii^—Cd1—Br1^i^	87.157 (14)
N1—Cd1—Br1^i^	85.92 (10)	Br1—Cd1—Br1^i^	92.843 (14)

Symmetry codes: (i) 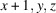; (ii) 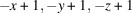.

**Table 2:** Hydrogen-bond geometry (Å, °)

*D*—H⋯*A*	*D*—H	H⋯*A*	*D*⋯*A*	*D*—H⋯*A*
C2—H2⋯Br1	0.95	3.03	3.604 (5)	120
C3—H3*B*⋯Br1^iii^	0.98	2.80	3.739 (6)	162

Symmetry code: (iii) 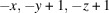.

**Table 3:** Experimental details

Crystal data	
Chemical formula	[CdBr_2_(C_6_H_8_N_2_)]
*M*_r_	380.36
Crystal system, space group	Monoclinic, *P*2/*c*
Temperature (K)	170
*a*, *b*, *c* (Å)	3.9508 (2), 7.5573 (4), 15.4616 (9)
β (°)	94.841 (5)
*V* (Å^3^)	460.00 (4)
*Z*	2
Radiation type	Mo *K*α
μ (mm^−1^)	11.00
Crystal size (mm)	0.14 × 0.10 × 0.08

Data collection	
Diffractometer	IPDS2
Absorption correction	Numerical (*X-RED* and *X-SHAPE*; Stoe, 2002 ▸)
*T*_min_, *T*_max_	0.124, 0.286
No. of measured, independent and observed [*I* > 2σ(*I*)] reflections	905, 905, 816
(sin θ/λ)_max_ (Å^−1^)	0.616

Refinement	
*R*[*F*^2^ > 2σ(*F*^2^)], *wR*(*F*^2^), *S*	0.034, 0.089, 1.09
No. of reflections	905
No. of parameters	54
H-atom treatment	H-atom parameters constrained
Δρ_max_, Δρ_min_ (e Å^−3^)	1.02, −0.79

Computer programs: *X-AREA* (Stoe, 2008 ▸), *SHELXT2014/4* (Sheldrick, 2015*a* ▸), *SHELXL2016/6* (Sheldrick, 2015*b* ▸), *DIAMOND* (Brandenburg, 1999 ▸), *XP* in *SHELXTL-PC* (Sheldrick, 2008 ▸) and *publCIF* (Westrip, 2010 ▸).

## supplementary crystallographic information

### Poly[di-µ-bromido-µ-2,3-dimethylpyrazine-κ2N1:N4-cadmium(II)] Crystal data

**Table:**

[CdBr_2_(C_6_H_8_N_2_)]	*F*(000) = 352
*M**_r_* = 380.36	*D*_x_ = 2.746 Mg m^−^^3^
Monoclinic, *P*2/*c*	Mo *K*α radiation, λ = 0.71073 Å
*a* = 3.9508 (2) Å	Cell parameters from 8176 reflections
*b* = 7.5573 (4) Å	θ = 1.3–26.7°
*c* = 15.4616 (9) Å	µ = 11.00 mm^−^^1^
β = 94.841 (5)°	*T* = 170 K
*V* = 460.00 (4) Å^3^	Block, colorless
*Z* = 2	0.14 × 0.10 × 0.08 mm

### Poly[di-µ-bromido-µ-2,3-dimethylpyrazine-κ2N1:N4-cadmium(II)] Data collection

**Table:**

IPDS-2 diffractometer	816 reflections with *I* > 2σ(*I*)
ω scans	θ_max_ = 26.0°, θ_min_ = 2.6°
Absorption correction: numerical (X-Red and X-Shape; Stoe, 2002)	*h* = −4→4
*T*_min_ = 0.124, *T*_max_ = 0.286	*k* = −9→9
905 measured reflections	*l* = −1→18
905 independent reflections	

### Poly[di-µ-bromido-µ-2,3-dimethylpyrazine-κ2N1:N4-cadmium(II)] Refinement

**Table:**

Refinement on *F*^2^	0 restraints
Least-squares matrix: full	Hydrogen site location: inferred from neighbouring sites
*R*[*F*^2^ > 2σ(*F*^2^)] = 0.034	H-atom parameters constrained
*wR*(*F*^2^) = 0.089	*w* = 1/[σ^2^(*F*_o_^2^) + (0.0624*P*)^2^] where *P* = (*F*_o_^2^ + 2*F*_c_^2^)/3
*S* = 1.09	(Δ/σ)_max_ = 0.001
905 reflections	Δρ_max_ = 1.02 e Å^−^^3^
54 parameters	Δρ_min_ = −0.79 e Å^−^^3^

### Poly[di-µ-bromido-µ-2,3-dimethylpyrazine-κ2N1:N4-cadmium(II)] Special details

**Table:**

Geometry. All esds (except the esd in the dihedral angle between two l.s. planes) are estimated using the full covariance matrix. The cell esds are taken into account individually in the estimation of esds in distances, angles and torsion angles; correlations between esds in cell parameters are only used when they are defined by crystal symmetry. An approximate (isotropic) treatment of cell esds is used for estimating esds involving l.s. planes.
Refinement. Refined as a 2-component twin.

### Poly[di-µ-bromido-µ-2,3-dimethylpyrazine-κ2N1:N4-cadmium(II)] Fractional atomic coordinates and isotropic or equivalent isotropic displacement parameters (Å^2^)

**Table:**

	*x*	*y*	*z*	*U*_iso_*/*U*_eq_	
Cd1	0.500000	0.500000	0.500000	0.0371 (2)	
Br1	0.00584 (12)	0.73950 (6)	0.46734 (4)	0.0399 (2)	
N1	0.4662 (12)	0.4338 (5)	0.3390 (3)	0.0404 (9)	
C1	0.4814 (13)	0.2822 (6)	0.2952 (4)	0.0367 (10)	
C2	0.4840 (13)	0.5847 (6)	0.2938 (3)	0.0400 (12)	
H2	0.474305	0.694575	0.323386	0.048*	
C3	0.4525 (16)	0.1113 (7)	0.3437 (4)	0.0443 (11)	
H3A	0.287711	0.033896	0.311400	0.066*	
H3B	0.376217	0.135673	0.401232	0.066*	
H3C	0.674531	0.052770	0.350202	0.066*	

### Poly[di-µ-bromido-µ-2,3-dimethylpyrazine-κ2N1:N4-cadmium(II)] Atomic displacement parameters (Å^2^)

**Table:**

	*U*^11^	*U*^22^	*U*^33^	*U*^12^	*U*^13^	*U*^23^
Cd1	0.0374 (3)	0.0367 (3)	0.0372 (3)	0.00148 (18)	0.0033 (2)	0.00196 (18)
Br1	0.0384 (3)	0.0354 (3)	0.0460 (4)	0.00116 (19)	0.0042 (3)	0.0029 (2)
N1	0.044 (2)	0.036 (2)	0.041 (2)	0.0024 (18)	0.0038 (19)	−0.0008 (18)
C1	0.035 (2)	0.037 (2)	0.038 (3)	0.0003 (19)	0.0058 (19)	0.001 (2)
C2	0.049 (3)	0.036 (3)	0.035 (3)	0.000 (2)	0.002 (2)	−0.001 (2)
C3	0.053 (3)	0.040 (3)	0.040 (3)	−0.001 (2)	0.009 (3)	0.000 (2)

### Poly[di-µ-bromido-µ-2,3-dimethylpyrazine-κ2N1:N4-cadmium(II)] Geometric parameters (Å, º)

**Table:**

Cd1—N1	2.531 (4)	C1—C1^iv^	1.418 (11)
Cd1—N1^i^	2.531 (4)	C1—C3	1.503 (7)
Cd1—Br1^i^	2.6789 (5)	C2—C2^iv^	1.372 (10)
Cd1—Br1	2.6789 (5)	C2—H2	0.9500
Cd1—Br1^ii^	2.7741 (5)	C3—H3A	0.9800
Cd1—Br1^iii^	2.7741 (5)	C3—H3B	0.9800
N1—C1	1.335 (6)	C3—H3C	0.9800
N1—C2	1.342 (6)		
			
N1—Cd1—N1^i^	180.00 (4)	C1—N1—C2	117.3 (5)
N1—Cd1—Br1^i^	91.66 (10)	C1—N1—Cd1	131.9 (3)
N1^i^—Cd1—Br1^i^	88.33 (10)	C2—N1—Cd1	110.1 (3)
N1—Cd1—Br1	88.34 (10)	N1—C1—C1^iv^	120.9 (3)
N1^i^—Cd1—Br1	91.66 (10)	N1—C1—C3	118.4 (5)
Br1^i^—Cd1—Br1	180.0	C1^iv^—C1—C3	120.7 (3)
N1—Cd1—Br1^ii^	85.92 (10)	N1—C2—C2^iv^	121.8 (3)
N1^i^—Cd1—Br1^ii^	94.08 (10)	N1—C2—H2	119.1
Br1^i^—Cd1—Br1^ii^	87.157 (14)	C2^iv^—C2—H2	119.1
Br1—Cd1—Br1^ii^	92.843 (14)	C1—C3—H3A	109.5
N1—Cd1—Br1^iii^	94.08 (10)	C1—C3—H3B	109.5
N1^i^—Cd1—Br1^iii^	85.92 (10)	H3A—C3—H3B	109.5
Br1^i^—Cd1—Br1^iii^	92.843 (14)	C1—C3—H3C	109.5
Br1—Cd1—Br1^iii^	87.157 (14)	H3A—C3—H3C	109.5
Br1^ii^—Cd1—Br1^iii^	180.0	H3B—C3—H3C	109.5
Cd1—Br1—Cd1^v^	92.843 (14)		

Symmetry codes: (i) −*x*+1, −*y*+1, −*z*+1; (ii) *x*+1, *y*, *z*; (iii) −*x*, −*y*+1, −*z*+1; (iv) −*x*+1, *y*, −*z*+1/2; (v) *x*−1, *y*, *z*.

### Poly[di-µ-bromido-µ-2,3-dimethylpyrazine-κ2N1:N4-cadmium(II)] Hydrogen-bond geometry (Å, º)

**Table:**

*D*—H···*A*	*D*—H	H···*A*	*D*···*A*	*D*—H···*A*
C2—H2···Br1	0.95	3.03	3.604 (5)	120
C3—H3*B*···Br1^iii^	0.98	2.80	3.739 (6)	162

Symmetry code: (iii) −*x*, −*y*+1, −*z*+1.
